# Neutrophils assist the metastasis of circulating tumor cells in pancreatic ductal adenocarcinoma: A new hypothesis and a new predictor for distant metastasis: Erratum

**DOI:** 10.1097/MD.0000000000006215

**Published:** 2017-02-17

In the article “Neutrophils assist the metastasis of circulating tumor cells in pancreatic ductal adenocarcinoma: A new hypothesis and a new predictor for distant metastasis”,^[1]^ the legends for Figures 1–3 were misplaced. The legend under Figure 1 should be associated with Figure 3. The legend under Figure 2 should be associated with Figure 1. The legend under Figure 3 should be associated with Figure 2.
